# Muscle energetics and the cardiovascular response to isometric exercise and post-exercise circulatory occlusion following exercise-induced muscle damage: insights from multiparametric MRI

**DOI:** 10.3389/fphys.2025.1636964

**Published:** 2025-10-29

**Authors:** Fabio Zambolin, Jean-Christophe Lagacé, Susan Pinner, James McStravick, Fiona E. Smith, Aneurin J. Kennerley, Jamie S. McPhee

**Affiliations:** ^1^ Department of Sport and Exercise Sciences, Manchester Metropolitan University, Manchester, United Kingdom; ^2^ Manchester Metropolitan University Institute of Sport, Manchester Metropolitan University, Manchester, United Kingdom; ^3^ Faculty of Physical Activity Sciences, Université de Sherbrooke, Sherbrooke, QC, Canada; ^4^ Research Centre on Aging, Sherbrooke Geriatric Institute, Université de Sherbrooke, Sherbrooke, QC, Canada

**Keywords:** magnetic resonance spectroscopy, muscle oxygenation, muscle metabolism, group III–IV muscle afferents, exercise-induced muscle damage, muscle inflammation, delayed onset of muscle soreness

## Abstract

**Introduction:**

Skeletal muscles damaged by exercise exhibit disturbed energy metabolism and microvascular function for several days. However, it remains unclear whether these local changes might affect systemic cardiovascular responses to exercise. The present study aimed to investigate whether damaged muscles show changes in energy metabolism and oxygenation that influence systemic cardiovascular responses to exercise and post-exercise circulatory occlusion (PECO).

**Methods:**

A novel multi-parametric magnetic resonance imaging and spectroscopy approach was applied. Twelve healthy male participants completed assessments before and 48 h after 40 min of downhill running (20% decline). The assessments included muscle function, inflammation, and multi-parametric imaging at rest, exercise, and post-exercise occlusion using ^31^P spectroscopy, ^1^H- and muscle blood oxygen level-dependent imaging, and ^23^Na^+^ imaging to assess phosphate metabolism, oxygenation, and sodium disturbances. The mean arterial pressure (MAP) and heart rate (HR) were recorded throughout the MRI sequences.

**Results:**

Forty-eight hours after downhill running, muscle inflammation and Na^+^ disturbances were evident (both *p* < 0.05). Muscle oxygenation was lower and inorganic phosphates were higher during exercise and PECO than at baseline (both *p* < 0.05). However, MAP and HR during exercise and PECO remained unchanged at 48 h compared with baseline.

**Conclusion:**

Our multi-parametric MRI approach provides new insights into the local effects of muscle damage on energy metabolism, oxygenation, and Na^+^. Despite these local metabolic and microvascular disturbances, systemic cardiovascular responses, as indicated by MAP and HR, remained unchanged. These new findings suggest a dissociation between muscle metabolites, oxygenation, and the cardiovascular response to exercise and PECO 48 h after damaging exercise.

## Introduction

Supporting exercising skeletal muscles by increasing blood flow while reducing metabolite accumulation is crucial for minimizing fatigue and preventing exercise intolerance (Amann et al., 2011). This requires a redistribution of the cardiac output, primarily to the exercising muscle mass, which is regulated in part by muscle nerve afferents and the central nervous system (Amann et al., 2020; Taylor et al., 2016). In this context, the role of feedback is pivotal and driven by two types of skeletal muscle nerve afferents: myelinated group III (Aδ) and unmyelinated group IV (C-fiber). These afferents detect mechanical and metabolic stimuli, respectively, to match cardiovascular adjustments with local muscle metabolic demands during exercise (Goodwin et al., 1972; McCloskey and Mitchell, 1972).

Afferent dysfunction has been reported in chronic inflammatory conditions, but it can also occur after acute, localized muscle damage (Matsubara et al., 2019; Teixeira and Vianna, 2022). However, the underlying mechanisms causing vascular dysfunction remain unclear. Previous studies have used exercise-induced muscle damage (EIMD) as a controlled inferential model to investigate the mechanisms underlying afferent-mediated changes in healthy populations (Lee et al., 2023; Zambolin et al., 2022a; Zambolin et al., 2024b; Zambolin et al., 2023).

EIMD is characterized by localized inflammation (Höger et al., 2023), muscular weakness, and soreness, and it usually peaks at 24 h–48 h post-exercise (Byrne et al., 2004). Additionally, EIMD is associated with increased phosphate muscle metabolism, ion perturbation (Gast et al., 2023), and impaired microvascular function (Caldwell et al., 2016; Davies et al., 2011; Kano et al., 2005; Larsen et al., 2019). However, although these physiological alterations are well-known, the interaction between muscle afferents, metabolism, and cardiovascular regulation in response to exercise and post-exercise circulatory occlusion (PECO) post-EIMD remains poorly understood. EIMD alters local metabolic and inflammatory responses, which may, in turn, affect the muscle metaboreflex. This reflex helps match the cardiovascular output to metabolic demand. However, the extent to which EIMD disrupts this coupling remains unclear. Previous studies suggest that EIMD alters afferent sensitivity, particularly in mechano- and nociceptive receptors, which may lead to exaggerated cardiovascular responses to passive leg movements and reduced hemodynamic regulation (Zambolin et al., 2022a). However, some studies report intact blood pressure (BP) responses in the presence of central command activity (Ray et al., 1998), while others demonstrate that the BP response during isometric exercise after eccentric exercise was enhanced, even when the workload on the damaged arm was reduced to match the perceived effort (i.e., central command) with the control arm (Miles et al., 1997). On the other hand, an increase in the central command to the extent required to compensate for eccentric-induced muscle weakness does not affect the ventilatory response during dynamic exercises (Hotta et al., 2016). Other studies have also reported reduced or intact metaboreflex responses during PECO at 24 h and 48 h post-EIMD (Lee et al., 2023; Zambolin et al., 2023). These discrepancies may arise due to methodological differences across the studies that cause different levels of muscle oxygenation and energy metabolic changes, both of which could significantly influence BP regulation during exercise and PECO (Lee et al., 2023). Potential methodological differences across studies may be related to the exercise load used to assess cardiovascular responses during exercise and PECO, both before and after EIMD. Some studies have utilized the same absolute workload pre- and post-EIMD, while others have reduced the post-EIMD workload to account for increased central command because of muscle soreness or weakness. However, adjusting workload intensity can alter muscle energetics and the metabolic environment—particularly oxygenation and phosphate metabolism—both at baseline and following EIMD. These changes may vary between individuals and influence cardiovascular responses independently of afferent feedback.

To address this, we chose to maintain the same absolute workload across conditions while controlling for metabolic activation by matching the phosphocreatine (PCr) depletion levels at baseline. This approach allowed us to standardize exercise intensity and contextualize cardiovascular responses to exercise and PECO in relation to muscle metabolic changes induced by EIMD.

Therefore, the present study aimed to investigate how post-EIMD changes in muscle energy metabolism and oxygenation influence cardiovascular response during isometric exercise and PECO. We hypothesized that EIMD would increase muscle energy metabolism (inorganic phosphate) and reduce oxygenation, which would lead to a concomitant increase in the cardiovascular responses during exercise and PECO. By directly linking these variables to cardiovascular responses during sustained exercise in weakened muscles and the presence of inflammation, this study provides new insights into pathophysiological mechanisms leading to altered cardiovascular function.

## Methods

### Participants

The study received ethical approval from the Faculty of Science and Engineering Research Ethics and Governance Committee (reference number: 48097) and was conducted in accordance with the Declaration of Helsinki, except for pre-registration. Twelve healthy young men (age, 25.2 ± 5.0 years; mass, 76.3 ± 11.4 kg; and height, 178.0 ± 7.6 cm) completed the study. One participant dropped out of the study due to sickness, and only six participants completed the ^31^P assessment because of time constraints and scanner-associated costs.

All participants abstained from caffeine and food for 2 hours prior to participation and avoided alcohol intake and intense exercise for 2 days prior to participation in the study. The exclusion criteria included the use of non-steroidal anti-inflammatory medication and the presence of any injury or medical conditions that prevented downhill running exercise. Participants were also excluded from MRI if they had a cardiac pacemaker or any other standard contraindications. Due to the nature of the study design, participants were not randomized, and blinding was not implemented.

### Experimental design

Participants attended the research laboratory for three visits. A familiarization session was conducted, during which a verbal explanation of the study procedures was provided, written informed consent was obtained, participants were familiarized with the MRI facility, and knee-extension assessments and appointments were scheduled for the first (pre-EIMD) and follow-up experimental sessions. Follow-up took place 48 h after baseline (post-EIMD). Pre- and post-EIMD assessments followed the same procedures. The EIMD exercise protocol was completed only once, at the end of the first session.

### Questionnaires and soreness assessment

A physical activity readiness questionnaire (PAR-Q) was completed, and standing height and body mass were measured. Perceived muscle soreness of the knee extensors was measured using a visual analog scale as the participants held a squat with the knees bent at 90˚ (VAS_SQ_) (Burt et al., 2012). The assessment was performed by asking participants to mark an “X” on a 10-cm scale to indicate the level of soreness: with 0 representing no muscle soreness and 10 representing muscles so sore that movement is impossible (Burt et al., 2012; Twist and Eston, 2009).

### Maximal voluntary contraction assessment

The participants sat upright on a Kineo Multistation Dynamometer (Globus Italia, Treviso, Italy) with the hips and knees flexed at 90˚ and straps secured around the waist to minimize extraneous movements. Single-leg maximal voluntary knee extension isometric contraction (MVC) was tested on the dominant limb, and real-time feedback of force was recorded. The dominant leg was determined via self-report, based on the preferred leg used for kicking a ball. A short warm-up was permitted consisting of 10 brief isometric contractions, increasing progressively from approximately 50% to 80% maximal effort. After 1 min of rest, participants performed three MVCs, each separated by 1 min of rest. Each MVC trial lasted 3 s–5 s, with verbal encouragement provided to ensure the maximal effort. The highest external force value was accepted as each participant’s MVC (Mcphee et al., 2014). This procedure was used to assess the initial load to be used for the exercise protocol that was later completed within the magnetic resonance (MR) scanner. For the MRI exercise load, a sandbag weighing 3 kg–5 kg was strapped firmly close to the ankle malleoli. The load was individualized depending on the initial MVC, allowing similar metabolism activation across participants, with a 20% ± 2% PCr reduction from baseline to the end of exercise. Standardization of PCr depletion between the participants was achieved by altering the ankle weight to a fraction of their MVC such that m = 0.131 MVC/(gּּּּL), where m is the ankle weight in kg, MVC is the torque in Nm, g is 9.81 ms^−2^, and L is the leg length in meters, which is defined as the distance from the lateral femoral condyle to the malleolus (Sleigh et al., 2016). Specifically, participants with 177 Nm–204 Nm MVC used a 3-kg sandbag, participants with 204 Nm–235 Nm MVC used a 3.5-kg sandbag, participants with 235 Nm–267 Nm MVC used a 4-kg sandbag, participants with 267 Nm–298 Nm MVC used a 4.5-kg sandbag, and participants with 298 Nm–330 Nm MVC used a 5-kg sandbag. The mean ± SD sandbag load was 4 ± 1 kg. Pre- and post-EIMD loads were kept the same for each individual participant.

### Quantitative multi-parametric MRI protocol

A Siemens MAGNETOM Vida 3 Tesla MRI system (Siemens Healthcare GmbH, Erlangen, Germany, housed in the university’s Wolfson ACTIVE Laboratory) was used for imaging/spectroscopy assessments of the thigh muscles. Participants were positioned feet-first supine, and all MRI sequences were centered on the mid-thigh, spanning the superior border of the patella to approximately 10 cm distal to the greater trochanter. The participants were provided with hearing protection, and the total time spent in the scanner, including the setup, was approximately 80 min. An integrated RF body coil was used for ^1^H signal transmission. A receive-only 16-channel ^1^H flex coil covered both thighs for structural imaging of the quadriceps. T1-weighted images (TR 800 ms, TE 12 ms) were acquired across 28 slices (0.5 × 0.5 × 7 mm voxels). T_2_ mapping utilized a 2D multi-spin-multi-echo (MSME) sequence with 17 equidistant echoes (TE1/ΔTE = 10 ms; TR = 3,000 ms; slice thickness = 5 mm; slice gap = 10 mm) (Azzabou et al., 2015). Thereafter, the ^1^H flex coil was removed, and a dual-tuned single-channel ^1^H/^31^P surface coil (^31^P/^1^H Flex Coil 3T, RAPID Biomedical GmbH) was placed on the anterior thigh of the dominant leg. The knee was raised to rest on a firm bolster 14.5 cm high (thus, flexing the hip to approximately 20˚ and placing the RF coil at the MRI isocenter to maximize the signal-to-noise ratio) (Meyerspeer et al., 2020). Sandbags (weight matching the relative load MVC assessment results) were strapped firmly around the distal tibia close to the malleoli (adapted from Sleigh et al., 2016). An ^1^H-based T_2_ localizer was acquired (3 slice packages; 15 slices; 256 mm^2^ field of view (FOV); 6 mm slice thickness; repetition time (TR) 606 ms; echo time (TE) 122 ms; number of averages (NA) 1; flip angle (FA) 150°; 60% phase resolution; 6/8 phase partial Fourier; 592 Hz/px; echo spacing 4.06 ms; turbo factor 154; RF pulse type, fast; and gradient mode, fast). This tri-planar localizer was used to position a cuboid adjustment window covering the entire thigh (the size and position covering the visible area of the individual thigh). Manual frequency, power, and 3D shimming adjustments were completed to achieve a ^1^H water peak with full width at half maximum (FWHM) of 19.9 ± 1.0 Hz and a ^31^P PCr peak with FWHM of 16.5 ± 0.97. 3D shimming utilized a multiple-gradient echo field mapping approach. The PCr resonance was set to 0 ppm. This was confirmed by a short ^31^P FID-based acquisition (TR 4,000 ms; NA 32; FA 90°; bandwidth (BW) 4,000 Hz; acquisition duration 512 ms; and spectral points 2,048). Scan parameters were as follows: NA = 32; TR = 12,000 ms; BW = 3,000 Hz; data points = 2,048; and acquisition duration = 692 ms. ^31^P spectra were acquired across the four workloads of the exercise protocol, including a 2-min of resting period, a 3-min submaximal sustained isometric contraction of the knee extensors, maintaining the shank with the ankle load parallel to the scanning bed, 2 min of PECO, and a 3-min resting recovery. At the beginning and end of the sustained knee extension contractions, participants were asked to provide their rating of perceived exertion (RPE) (Williams, 2017). Following these procedures, the participants rested for 10 min–15 min outside the MRI scanner, while a flexible coil (^23^Na/^1^H Flex Coil 3T, RAPID Biomedical GmbH) was placed on the anterior thigh of the dominant leg. The participants then returned to the scanner to repeat the knee extension exercise. 2D radial FLASH-based ^23^Na images were captured pre- and post-exercise (TR 50 ms; TE 2.3 ms; FOV 250 mm^2^; matrix size 64^2^; flip angle 90^o^; and thickness 100 mm). Echo planar-based blood-oxygen-level-dependent muscle oxygenation (mBOLD) acquisition was performed every 1 s (TR = 2 s) for a total of 10 min to capture rest, sustained isometric knee extension exercise, PECO, and recovery workloads (as described above).

### Cardiovascular assessment and post-exercise cuff occlusion protocol

During the MRI exercise protocols, participants wore a beat-by-beat, non-invasive MRI-compatible blood pressure plethysmograph (Biopac System, United Kingdom) to monitor the mean arterial blood pressure (BP) and heart rate (HR). MAP and HR were collected continuously during the ^31^P MR spectroscopy and BOLD acquisition protocols. PECO used a rapid inflatable cuff (13 cm wide; E. Hokanson Inc., Bellevue, WA 98005, United States) around the proximal thigh. The cuff was inflated with compressed air to 250 mmHg–270 mmHg 15 s before the end of the 3-min sustained leg extension contraction. After 2 min, the cuff was instantly deflated. Arterial and venous occlusion was confirmed by the absence of a posterior tibial artery pulse (Patterson et al., 2019).

### Exercise-induced muscle damage protocol

The EIMD protocol consisted of 40-min downhill running (DHR) at 20% decline on a treadmill (HP Cosmos Saturn 300/100, Nussdorf-Traunstein, Germany). The treadmill speed was set at 10 km/h, and a 5-min warm-up was provided as the treadmill gradient was gradually changed from 0% to 20% decline (Bontemps et al., 2020). The treadmill speed, HR, mechanical power, and RPE were measured at 5 min intervals throughout the 40-min run. The treadmill speed was reduced to 8 Km/h if the participants reported RPE of 18 or asked for a slower running speed. A 2-min cool-down period at 0% decline at a self-selected speed was provided after the 40-min run.

### Data analysis and MRI data processing

All MRI data (QCSA, T_2_ maps, muscle oxygenation, ^31^P and ^23^Na) were analyzed in MATLAB (2020b; MathWorks, Natick, MA, United States) using software routines developed in-house and available upon reasonable request. EIMD outcomes and cardiovascular responses to exercise were reported in Excel (Microsoft, Redmond, Washington, United States). Cardiovascular, muscle metabolism, and oxygenation metrics were analyzed for each given workload. Changes in sodium concentration were calculated by subtracting the resting values from post-exercise values for each time-point (pre-EIMD and post-EIMD).

For imaging data, a region of interest (ROI) was placed over the thickest portion of the quadriceps muscles (i.e., *vastus lateralis*, *vastus intermedium*, *rectus femoris*, *and vastus medialis*), avoiding subcutaneous fat and bone. The ROI mask was used to evaluate the quadriceps cross-sectional area (QCSA) on the T_1_-weighted image. Using the MSME images, quantitative muscle-water T_2_ maps were reconstructed based on a tri-exponential fitting procedure (Azzabou et al., 2015; Reyngoudt et al., 2022). Muscle-water T_2_ values are reported as the mean from an ROI covering the interior of the *vastus lateralis* muscle. Muscle oxygenation (mBOLD) was analyzed from the imaging slice with the maximum QCSA. The *vastus lateralis* was used as the reference, motion artifacts were removed (using image registration), and the oxygenation signal was recorded and expressed as fold changes from rest. ^23^Na datasets were reconstructed offline using a non-uniform fast Fourier transform and calculated across. Before co-registration to the ^1^H images (tri-pilot images), a correction for gradient nonlinearities was performed. Then, a region-based partial volume correction using the tissue masks was applied to the ^23^Na images. Finally, sodium total concentration values were determined using the corrected signal intensities based on three external references (NaCl at 10/20/30 mmol). For ^31^P spectral data, raw frequency data underwent 5 Hz line broadening (Gaussian filter) to improve SNR. Spectra were manually phased (zeroth- and first-order correction). Following the Fourier transform, the baseline was fitted to a fourth-order polynomial and removed (elevated due to phospholipid contributions). The resultant spectral peaks were assigned to the following ^31^P resonances: phosphomonoesters (PMEs), inorganic phosphate (Pi; intracellular ∼4.9 ppm and extracellular ∼5.3 ppm), phosphodiesters (PDEs), phosphocreatine (PCr), and the three resonances of adenosine triphosphate (γ-ATP, α-ATP, and β-ATP). To quantify signal contributions from intracellular inorganic phosphate (Pi(i)), the spectra were further windowed to 4 ppm–6 ppm. Data were fitted using nonlinear least squares with a Levenberg–Marquardt algorithm to appropriate Gaussian/Lorentzian functions (to minimize the residual) for Pi(i) and Pi(e) with chemical shift, amplitude, and FWHM floating variables. Fitting parameters were used to isolate the signal contribution from intracellular Pi. The area under the curve (AUC) was extracted as a function of phase and time-point (averaged across the collected spectra). All data were normalized to the resting phase across the functional task.

### Statistical analysis

Power analysis for sample size calculation was determined on the difference between pre- and post-changes in muscle oxygenation, as the primary outcome, reported by Larsen et al. (2019). Results from power calculation analysis using two-way repeated measures analysis of variance (ANOVA) for two groups were as follows: λ = 12.20, F = 4.9, and actual power = 0.87. This resulted in an estimated total sample size of n = 12 participants. While some secondary outcomes had smaller sample sizes (e.g., ^31^P MRS, n = 6), Cohen’s d effect sizes are reported to aid interpretation. Statistical analysis was carried out in GraphPad Prism (v.10.1 GraphPad software, San Diego, California United States). The normal distribution of the data was assessed using a Shapiro–Wilk test. If the sphericity assumption was violated, the Greenhouse–Geisser correction coefficient was reported. A paired t-test was performed for pre- and post-EIMD muscle soreness and neuromuscular outcomes (i.e., VAS_SQ_, MVC, QCSA, T_2_ maps, resting [Na^+^], and delta [ΔNa^+^] concentrations). A two-way repeated measures ANOVA was performed for muscle oxygenation (mBOLD), Pi, Pi(i), Pi(e), MAP, and HR for workload (rest, contraction, PECO, and recovery) and time (pre-EIMD vs. post-EIMD). For all these variables, Cohen’s d effect sizes (ESs) were calculated as delta differences between delta changes from rest to exercise (Δexercise) and rest to PECO (ΔPECO) between pre- and post-EIMD measures by the pooled SD for each delta change. Cohen’s d effect size thresholds were considered at 0.2, 0.5, and 0.8 for small, moderate, and large effect sizes, respectively (Higgins and Green, 2008).

## Results

### Neuromuscular function, soreness, and sodium concentrations following EIMD

**FIGURE 1:**
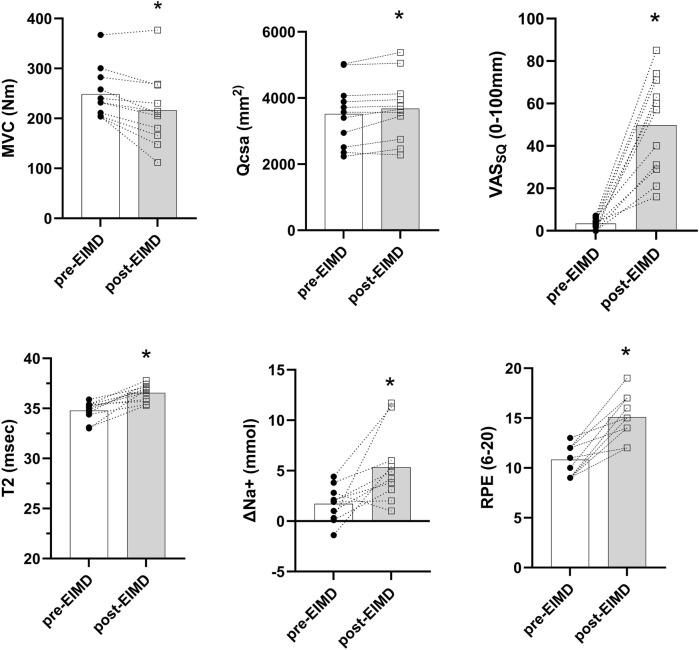
Markers of EIMD before and 48 h after downhill running. MVC, maximal voluntary contraction; VAS_SQ_, visual analog scale for soreness; T_2_, 1H T_2_ map; QCSA, quadriceps cross-sectional area; ΔNa^+^, delta sodium from rest to exercise; RPE, rate of perceived exertion. The bars represent the group means, and individual data points are also shown; *p < 0.05.

Neuromuscular function, soreness, inflammation, and sodium concentrations before and after EIMD are summarized in Figure 1. The downhill running protocol significantly impaired neuromuscular function, with a decrease in MVC torque (pre-EIMD: 248.5 ± 51.2 Nm vs. post-EIMD: 216.1 ± 71.3 Nm; *p* < 0.01; ES = 0.52) and an increase in QCSA (pre-EIMD: 3518 ± 965 mm^2^ vs. post-EIMD: 3675 ± 970 mm^2^; p < 0.01; ES = 0.16) and VAS_SQ_ (pre-EIMD: 4.9 ± 4.9 mm vs. post-EIMD: 49.7 ± 23.3 mm; *p* < 0.01; ES = 2.6), indicating inflammation and tissue swelling. Consistent with these findings, muscle-water T_2_ mapping values increased significantly post-EIMD (pre-EIMD: 34.8 ± 0.9 ms vs. post-EIMD: 36.5 ± 0.8 ms; *p* < 0.01; ES = 1.99). The resting sodium concentration (Na^+^) did not change significantly (pre-EIMD: 29.8 ± 5.9 mmol vs. post-EIMD: 31.5 ± 6.2 mmol; *p* = 0.98; ES = 0.28). However, ΔNa^+^ (change in sodium from rest to post-exercise) was significantly increased post-EIMD (pre-EIMD: 1.7 ± 1.8 mmol vs. post-EIMD: 5.3 ± 3.6 mmol; *p* < 0.01; ES = 1.26), indicating a greater ionic imbalance during the sustained isometric contraction. Additionally, RPE during the exercise contraction inside the scanner was significantly higher post-EIMD (pre-EIMD: 14.0 ± 2.1 vs. post-EIMD: 18.3 ± 1.2; *p* < 0.01; ES = 2.5).

### Muscle oxygenation and metabolism responses following EIMD

**FIGURE 2:**
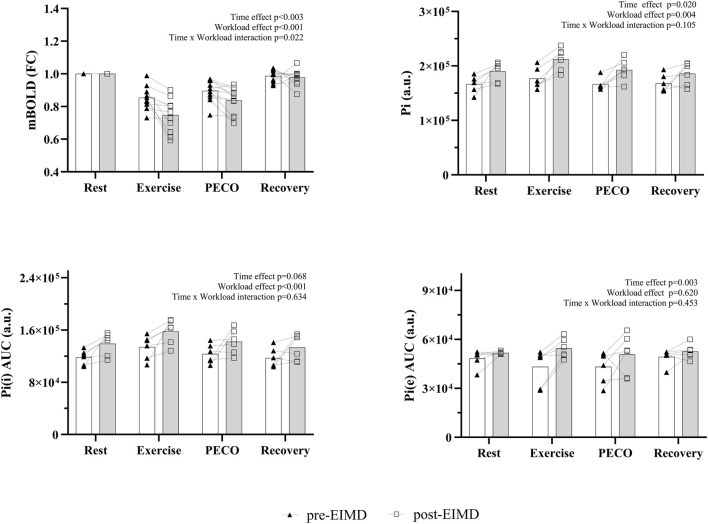
Changes in muscle energy metabolism and oxygenation before (pre-EIMD) and 48 h after EIMD (post-EIMD). Rest, measurements taken at rest; PECO, post-exercise circulatory occlusion; AUC, area under the curve; mBOLD, muscle blood oxygenation level-dependent; Pi(i), intracellular phosphate; Pi(e), extracellular phosphate; a.u, arbitrary units; FCs, fractional changes. The bars represent the group means, and individual data points are also shown.

**FIGURE 3:**
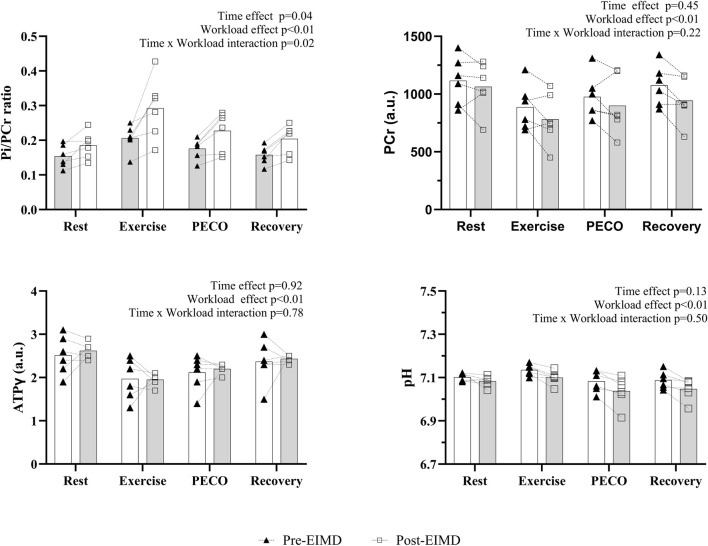
Changes in muscle energy metabolism before (pre-EIMD) and 48 h after EIMD (post-EIMD). Rest, measurements taken at rest; PECO, post-exercise circulatory occlusion; AUC, area under the curve; ATP, adenosine triphosphate; PCr, phosphocreatine; Pi, inorganic phosphate; a.u, arbitrary units; FCs, fractional changes. The bars represent the group means, and individual data points are also shown.

Muscle oxygenation and energy metabolism before and after EIMD are shown in Figures 2, 3. The reduction in muscle oxygenation (mBOLD) that occurred during exercise and PECO was greater for post-EIMD measurements than for pre-EIMD measurements [(workload effect: *p* < 0.01; F_(1,20)_ = 39.41); time effect (*p* = 0.03; F_(2,37)_ = 4.88); and time * workload interaction (*p* = 0.02; F_(3,60)_ = 3.53)].

^31^P data were available from six participants. Pi at rest was significantly elevated post-EIMD compared with pre-EIMD but showed similar relative increases with exercise and PECO in the pre- and post-EIMD states (*p* < 0.01; workload effect: F_(1,10)_ = 14.21; time effect: *p* = 0.02; F_(2,20)_ = 6.63; and time * workload interaction: *p* = 0.17; F_(3,30)_ = 1.78). Further analysis revealed that intracellular Pi concentration increased significantly with workload (*p* < 0.01; F_(2,16)_ = 22.63), with a trend for the effect of time (*p* = 0.068; F_(1,10)_ = 4.25). Extracellular Pi remained unchanged across workloads (*p* = 0.52; F_(2,20)_ = 0.66) but increased significantly post-EIMD (*p* = 0.03; F_(1,10)_ = 6.24). The Pi/PCr ratio showed a significant effect of workload (*p* < 0.01; F_(2,17)_ = 30.92), time (*p* = 0.048; F_(1,10)_ = 6.83), and time * workload interaction (*p* = 0.020; F_(3,30)_ = 3.53). ATPγ, PCr, and pH values showed significant changes over phase (ATPγ: *p* < 0.01, F_(2,23)_ = 39.50; PCr: *p* < 0.01, F_(1,16)_ = 58.77; and pH: *p* < 0.01, F_(1,13)_ = 15.83) with no changes in time (ATPγ: *p* = 0.75, F_(1,10)_ = 0.10; PCr: *p* = 0.45, F_(1,10)_ = 0.60; and pH: *p* = 0.12; F_(1,10)_ = 2.73) or time * workload interaction (ATPγ: *p* = 0.77; F_(3,30)_ = 0.37; PCr: *p* = 0.22, F_(3,30)_ = 1.45; pH: p = 0.51, F_(3,30)_ = 0.78). The corresponding effect sizes reporting changes in Δexercise and ΔPECO between pre- and post-EIMD values are reported in Figure 5.

### Cardiovascular responses following EIMD

**FIGURE 4:**
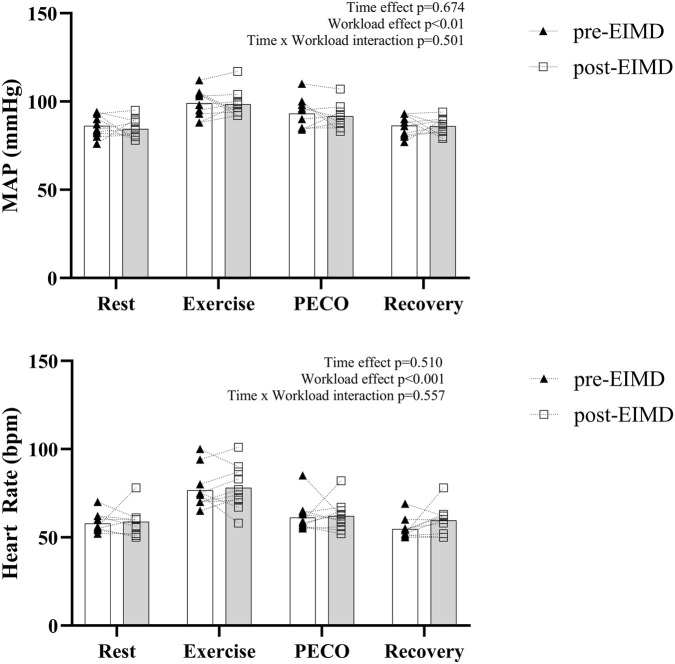
Changes in cardiovascular responses before and 48 h after EIMD. Rest, resting; PECO, post-exercise circulatory occlusion; MAP, mean arterial blood pressure. The results from the statistical analysis and its respective *p*-values are reported on the bottom right of each graph. The bars represent the group means, and individual data points are also shown.

**FIGURE 5:**
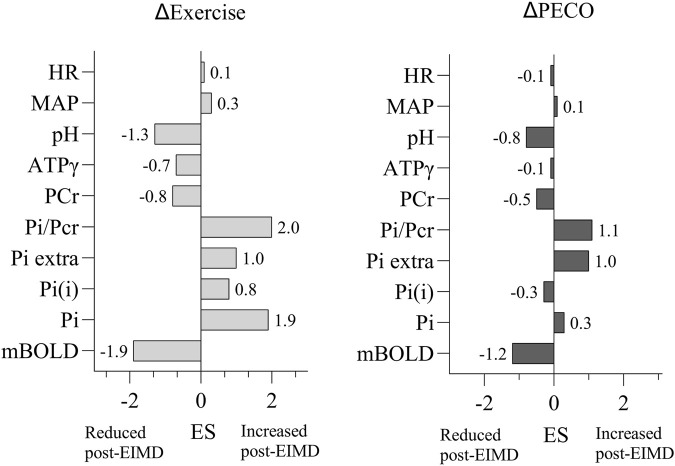
Effect sizes of delta exercise and delta PECO responses before and 48 h after EIMD. PECO, post-exercise circulatory occlusion; mBOLD, muscle blood oxygenation level-dependent; Pi(i), intracellular phosphate; Pi(e), extracellular phosphate; ATP, adenosine triphosphate; PCr, phosphocreatine; Pi, inorganic phosphate; MAP, mean arterial blood pressure. The results from the statistical analysis and its respective *p*-values are reported on the bottom right of each graph. The bars represent the group means, and individual data points are also shown.

Cardiovascular responses (MAP and HR) to exercise are shown in Figure 4. Both MAP and HR increased significantly with workload (*p* < 0.001), but no significant main effect of time or time * workload interaction was observed, suggesting that the autonomic cardiovascular response remained unchanged post-EIMD despite altered muscle oxygenation and energy metabolism. The corresponding effect sizes reporting changes in Δexercise and ΔPECO between pre- and post-EIMD values are reported in Figure 5.

## Discussion

This study provides the first accounts of the changes in muscle energy metabolism, oxygenation, and perceived exertion following EIMD and their relationship with the cardiovascular responses to exercise and PECO. This was achieved using a more comprehensive multi-parametric MRI approach. The primary novel findings were that damaged skeletal muscles exhibited lower oxygenation and higher Pi during exercise than in the non-damaged state, while MAP and HR were unchanged despite an increased RPE between pre- and post-EIMD. These results suggest that the systemic neuro-vascular regulation remains unaffected despite the substantial local muscle metabolic impairments and muscle weakness experienced by the participants that occurred following EIMD.

### Skeletal muscle function following EIMD

The downhill running protocol caused muscular weakness, tenderness, and inflammation when measured 48 h later (Figure 1), which is in line with the anticipated levels of EIMD following downhill running (Bontemps et al., 2020; Byrne et al., 2004). In this regard, we observed 14% lower MVC at 48 h, which is similar to a past study using downhill running protocols in young healthy individuals (Hayashi et al., 2019). We also observed an increase in muscle soreness (Chen et al., 2009) and inflammation (i.e., muscle-water T_2_ maps) (Holodov et al., 2023) and QCSA, indicating localized swelling (Whitehead et al., 1998; Whitehead et al., 2001). Inflammation accumulates extra fluid in the interstitial spaces, which increases the mobility of water molecules within the tissue, leading to a longer T_2_ relaxation time (Patten et al., 2003). Muscle damage after downhill running occurs as sarcomeres are forcibly lengthened under tension (Douglas et al., 2017), affecting extracellular structures, the sarcolemma, and myofibrils (McNeil and Khakee, 1992), leading to localized inflammation (Peake et al., 2017), metabolic disruption (Fouré et al., 2015), and possible changes to muscle afferent sensitivity and activation (Fujii et al., 2008).

### Sodium perturbation following EIMD

A novel finding of this study was the greater increase in muscle sodium concentration after a few minutes of damaged muscles, compared to the increase after exercising in non-damaged muscles than the increase after exercising non-damaged muscles. Na^+^ plays a critical role in maintaining action potential transmission during muscle contraction (Renaud et al., 2023). The failure to transport Na^+^ across the sarcolemma could contribute to excitation–contraction coupling failure, which may contribute to the loss of force production and increased muscle fatigue following eccentric contractions (Yeung et al., 2003).

Our findings build on the previous work that has shown increases in resting Na^+^ concentration 24 h following EIMD (Gast et al., 2023; Höger et al., 2023), correlating muscle damage markers (i.e., creatine kinase) with increases in Na^+^ concentrations. Changes in Na^+^ concentration have been attributed to the disruption of the sarcolemma, involving increased concentration of Na^+^, calcium ions (Stožer et al., 2020), and phosphate (Pi) (Kemp and Bevington, 1993). Na^+^ is expected to increase during exercise (2- to 2.5-fold during muscle contractions) (McKenna et al., 2008) and remain elevated following fatiguing exercise, where increases in Δ[Na^+^] are reported to be approximately 30% at 15 min following exercise with slow recovery rates (approximately 60 min) (Bansal et al., 2000). Unfortunately, we cannot discriminate changes occurring between intra- and extracellular Na^+^ with our MRI sequence, which would have been helpful in explaining the mechanism underlying these changes. However, our findings point toward a link between increased ion perturbation and increased muscle fatigue and weakness in damaged muscles.

### Muscle oxygenation following EIMD

By applying mBOLD sequences, we found a significant reduction in muscle oxygenation during exercise and PECO, which was greater for damaged muscles than for non-damaged muscles (Figure 2). These findings align with previous research in resting conditions (Caldwell et al., 2016), where impaired muscle oxygenation was noted 48 h following EIMD (Caldwell et al., 2016). However, our study provides new evidence of decreased oxygenation during exercise in the thigh muscles, which reflects a mismatch between local perfusion of oxygenated blood and local oxygen utilization. This may occur if damaged muscles utilize relatively more oxygen to hold the sustained isometric contraction without an increase in blood flow. Moreover, our results show that the relative increases in Pi and Pi/PCr from rest to exercise and PECO were greater in damaged than in non-damaged muscles, suggesting that energy turnover and metabolism differed between the two conditions, with a large effect size (Figures 2- 5). Another possible explanation is that the endothelial cells of the microvasculature were impaired due to changed circulation and impaired microvascular responses after muscle damage (Zambolin et al., 2022a). A link has been previously proposed between inflammation and endothelial cell impairments, which might be the underlying mechanism of an impaired microvascular response following EIMD (Stenvinkel, 2001).

### Interactions between muscle metabolism and the cardiovascular responses following EIMD

In line with our hypothesis, we observed significant changes in muscle energy metabolism (higher levels of Pi at rest and during exercise) and reduced oxygenation during exercise of damaged muscles. However, contrary to the second part of our hypothesis, we observed no change in cardiovascular responses (MAP and HR) during exercise and PECO following EIMD. This was surprising considering that an increase in phosphate metabolism and reduced pH has been linked to increased muscle sympathetic nerve activity and blood pressure response to exercise in non-damaged muscle (Boushel M. et al, 1998). In this regard, previous investigations examined the cardiovascular responses to exercise and the metaboreflex by applying PECO to assess the involvement of muscle nerve afferents following EIMD (Lee et al., 2023; Zambolin et al., 2023). PECO avoids any involvement of the central command while maintaining activation of muscle afferent activity (Alam and Smirk, 1937). These recent studies used relative loads, which meant that the load at 48 h was lower than that used for baseline conditions. They found contrasting results in MAP responses following EIMD. Lee et al. (2023) found attenuated MAP responses during exercise and PECO at 24 h, while in our previous work, no differences in MAP were found at 48 h (Zambolin et al., 2023). Therefore, it was suggested that these results might have been caused by the increased muscle weakness, the differences in workload used, or the changes in muscle energetics occurring following EIMD. However, in this current study, even after controlling for some of these variables, we found no differences in MAP and HR responses to exercise and PECO 48 h post-EIMD.

It is well-known that blood pressure increases with exercise intensity to help meet the higher demand for oxygen and nutrients by the muscles (Kaufman and Hayes, 2002; Zambolin et al., 2022b). The exercise pressor reflex, involving muscle nerve afferents, plays a crucial role in this process (Rowell, 1992). Our results might suggest that localized damage changes the relationship between muscle mechanoreceptor and metaboreceptor afferent activity, which normally increases the heart rate and blood pressure in proportion to the increase in metabolites and muscle stretch (Murphy et al., 2011). The change may be linked to inflammation changing the afferent sensitivity and responsiveness, which can, in turn, affect cardiovascular responses (Rotto et al., 1990). Inorganic phosphate accumulation during isometric contractions contributes to increased blood pressure by activating metaboreceptors, which trigger sympathetic responses (Boushel R. et al, 1998). However, in muscle inflammation models, the expected increase in blood pressure might not occur despite Pi accumulation due to factors such as altered afferent signaling, changes in endothelial function, and modulation of the autonomic nervous system (Westerblad et al., 2002). Our findings suggest a mismatch between cardiovascular responses and muscle Pi in damaged muscles as the increased Pi and Pi/PCr at rest and during exercise and PECO were not accompanied by corresponding increases in blood pressure (Sterns et al., 1991). This could be due to metabo-sensitive afferents becoming less responsive to metabolite accumulation, thus interfering with blood pressure regulation during exercise. Previous studies have found blunted metaboreflex activation in patients with chronic heart failure (Sterns et al., 1991), while others reported increases in mechanoreflex sensitization in response to cyclooxygenase (COX) but not lactic acid or adenosine produced during exercise (Middlekauff and Chiu, 2004; Middlekauff and Sinoway, 2007). EIMD is known to increase muscle inflammation and the production of prostaglandins and COX (Paulsen et al., 2010; Peake et al., 2017), but at the same time, it may also produce higher amounts of lactate, which has been hypothesized to reduce metaboreceptor sensitivity (Sterns et al., 1991). Moreover, recent research has found an increased mechanoreflex activation in response to static stretching following delayed onset muscle soreness (DOMS) in the absence of central command, suggesting mechano-nociceptors sensitization following EIMD (Zambolin et al., 2022a), while other studies found no changes or blunted metaboreflex responses to EIMD (Lee et al., 2023; Zambolin et al., 2023) when the central command was not activated.

Thus, following EIMD, there may be blunted metaboreflex activation with concomitant hyperactivation of the mechanoreflex, resulting in similar blood pressure responses during exercise and PECO. This mechanism may impact exercise performance as the increase in metabolic demand is not accompanied by a corresponding increase in blood pressure, oxygenation, or other factors contributing to the perception of effort and fatigue (Hureau et al., 2022; Zambolin et al., 2024a; Zambolin et al., 2024b). It is also important to clarify the relationship between the exercise pressor reflex (EPR)—a cardiovascular response mediated by group III/IV muscle afferents sensitive to mechanical and metabolic stimuli—and DOMS, which is typically associated with muscle damage and inflammation following eccentric exercise. Rather than continuous activation, eccentric exercise is thought to result in sensitization of these thin-fiber afferents, which may contribute to both DOMS and exaggerated reflex cardiovascular responses. Thus, the distinction between EPR and DOMS should not be viewed as a strict dichotomy as both phenomena involve overlapping afferent pathways. Prior work has questioned whether these represent two distinct classes of primary afferent neurons (Wenk and McCleskey, 2007). Our study did not directly assess nociceptive pathways, which may have influenced the cardiovascular responses observed, and this nuance should be considered when interpreting the findings.

### Methodological consideration and study limitations

Unfortunately, due to time constraints and the type of study design we adopted, we could not control for the lack of time-matched controls, making it impossible to exclude time effects unrelated to EIMD. However, this study design has been utilized quite extensively in the literature, and the fact that we controlled for major confounding factors (exercise, medications, and diet) makes our inferences potentially more accurate. Furthermore, the subset of six participants involved in the ^31^P experiments makes it difficult to draw conclusive arguments about the presence of differences despite the large changes that were also found in the effect size analysis (Williams et al., 2023). Nevertheless, our findings align with previous studies that have observed changes in muscle energetics following EIMD (Davies et al., 2011; Fouré et al., 2015). Additionally, our study acknowledges that some of the metabolites investigated, such as phosphate, might not contribute directly to the EPR, despite previous research in the field (Ducrocq and Kaufman, 2020; Sinoway et al., 1994). The increases in Pi observed might have been concomitant with exercise but not directly related to the EPR. Previous findings regarding the role of phosphate in the exercise pressor reflex might be over-interpreted, and the potential resetting of afferents at a higher operating point in response to acute alterations was not adequately addressed. However, we found a large effect size reduction in pH post-EIMD, which has been previously shown to be directly correlated with increased protons released and metaboreceptor activation (MacLean et al., 2000). Moreover, the observed increased in extracellular phosphate may represent a potential trigger for predominantly metabo-sensitive muscle nerve afferents (group IV) as the terminal endings of these afferents are located outside the cell (Mense, 2010; Stacey, 1969). However, future research needs to be conducted in this regard to confirm these assumptions. This study included only male participants to reduce variability related to hormonal fluctuations and sex-specific differences in muscle damage responses. However, this limits the generalizability of our findings, and future studies should include female participants to address potential sex-specific differences in EPR and muscle metabolism following EIMD.

Finally, the intensity of the exercise might not have been sufficiently intense to allow for a sufficient metabolite accumulation to trigger the metaboreflex. However, this intensity is still debatable, and no clear indication is present in the literature, making *a priori* assumptions in humans impossible. Based on our RPE data, we can assume that the intensity of the exercise, especially 48 h post EIMD, was increased, with a concomitant increase in muscle metabolism pointing toward the intense exercise domain.

## Conclusion

Our findings provide new insights into the relationship between muscle oxygenation, energy metabolism, and the cardiovascular response to exercise and PECO following EIMD. The novel multi-parametric imaging approach revealed changes in the relationships when damaged skeletal muscles were exercised. We found that oxygenation decreased to lower levels and Pi levels were elevated during exercise with EIMD. This corresponded with elevated perceptions of effort but with unchanged cardiovascular response to exercise and PECO from the baseline condition. These results suggest that the neuro-vascular regulation and blood pressure response to exercise become uncoupled from the peripheral skeletal muscle energetic disturbance with EIMD.

## Data Availability

The original contributions presented in the study are included in the article/supplementary material; further inquiries can be directed to the corresponding author.
